# Use of High Oleic Palm Oils in Fluid Shortenings and Effect on Physical Properties of Cookies

**DOI:** 10.3390/foods11182793

**Published:** 2022-09-10

**Authors:** Melissa Perez-Santana, Gloria B. Cagampang, Christopher Nieves, Victor Cedeño, Andrew J. MacIntosh

**Affiliations:** Food Science and Human Nutrition Department, University of Florida, Gainesville, FL 32611, USA

**Keywords:** high oleic palm oil, shortening, palm oil, differential scanning calorimetry, baking, solid fat content

## Abstract

Quality characteristics of bakery products rely partially on the amount and type of fats in their formulation. This study focused on producing emulsified shortenings with high oleic palm oil fractions to be thermo-mechanically characterized and used in the baking of high-fat cookies. Palm oil and hydrogenated fats were commonly used in bakery shortenings to achieve texture and flavor. However, saturated and trans-fats have been shown to cause detrimental health effects, motivating their replacement by unsaturated fats. High oleic palm oil (HOPO) is a novel oil with lower saturated fat and higher oleic acid compared to traditional palm oil (TPO). High oleic red olein (HORO) is a carotene-rich fraction of HOPO. Emulsified shortenings with 30% saturated fat containing HOPO, HORO, and TPO were produced. All shortenings resulted in similar onset temperatures of crystallization and melting points through DSC. Mid-melting peaks observed on TPO where absent in HOPO and HORO shortenings, reflected in lower hardness and calculated SFC of HOPO and HORO shortenings vs. TPO shortening. However, physical properties of shortening-containing cookies were not statistically different. It was demonstrated how HOPO and HORO can be used as alternative fats to TPO in the making of shortenings to be used in baking applications.

## 1. Introduction

In industrial baking applications, vegetable fats are mostly consumed in the form of shortenings or margarine [1]. These tailored fats are used because they contribute to the aeration and plasticity of dough [2] while also contributing to the texture, moisture retention, appearance, flavor, shelf-life and nutritional value of the final products [3]. This study examines for the first time the use of High Oleic Palm Oil as part of a tailored fat, specifically shortenings, compared to traditional palm oil. The physical properties of these shortenings were analyzed. The shortenings were then used in a baking application and effects on the physical properties of the products were compared.

In the late 20th century, a group of low cost baking fats were produced by partial hydrogenation from common oil sources such as soybean, sunflower or canola oil as reviewed by Ghotra et al. [4], the versatility of these fats supported the rise of numerous commercial brands of shortenings and margarines as compiled by Tarrago et al. [5]. Hydrogenation was often used to produce baking shortenings with typical values of 30% saturated fatty acids [6,7]. The desirable characteristics of baking shortenings include good plasticity i.e., preserving their semi-solid state at working temperatures, maintaining the functionality of aeration and texture for baked products even under thermal and mechanical stress, and having high oxidative stability [8]. However, partially hydrogenated fat consumption has recently been discouraged and limited due to the presence of trans fatty acids which are linked to increased risks of coronary heart disease [9]. Alternative methods to partial hydrogenation for shortening production with desired properties include blending with alternative oils such as rice bran oil, mahua oil [10], sal (*Shorea robusta*), illipe butter, bambangan kernel [11], mango kernel, kokum, shea, sunflower hard stearin (high oleic-high stearic) among others [12] and interesterification. 

The method of blending fats and oils is a low-cost option to produce baking fats with similar performance to partially and fully hydrogenated fat [12]. Emulsifier addition helps liquid fats to exert the same effect of plastic shortenings, with the benefit of easier handling on an industrial scale compared to solid shortenings [13]. Maruyama et al. [14] showed that mono- and di- glyceride emulsifiers contribute to the generation of elastic crystal networks when mixed with palm olein, increasing potential uses for this oil. Moreover, fluid shortenings have been successfully used in the formulation of cakes, bread, buns, rolls, and pie crust [15,16,17]. This is explained in the case of emulsified shortenings because emulsifiers shift towards the edge of the fat-air and fat-moisture interfaces [18], stabilizing the structure of the batter/dough during mixing, and later during baking [19]. 

Traditional palm oil (TPO) extracted from the palm *Elaeis guineensis* is one of the most consumed oils in the baking industry because it is mostly solid at room temperature without the need for hydrogenation [20]. This allows direct blending without additional processes to increase the firmness of the blend. However, the high content of saturated fat in palm oil and other edible fats is a concern for consumers because of increased risk of coronary heart disease [21]. 

High Oleic Palm Oil (HOPO), also known as Palm Oil Higher in Oleic Acid, is a newly available oil extracted from the hybrid palm *Elaeis oleifera x Elaeis guineensis*. High Oleic Red Olein (HORO) is a liquid fraction of HOPO that preserves a high proportion of the original pro-vitamin A carotenes and vitamin E tocols in the crude oil [22,23]. Tocols are referred to in this document as a naturally occurring group of lipid compounds with a methyl-substituted chromanol ring attached to a C16 isoprenoid chain [24]. However, to date, there is only one study in which HORO has been used in a baking-food application as a source of micronutrients and a health-contributing oil compared to TPO [25,26]. HOPO and HORO contain less saturated fat than TPO and super olein respectively and contain roughly equivalent increases in oleic acid [22,27]. The replacement of saturated fat by oleic acid has been correlated to lower risks of coronary heart disease through a decrease in total serum cholesterol and low-density lipoprotein-cholesterol [28,29]. Higher portions of oleic acid in HOPO and HORO make possible the formulation of tailored shortenings that can provide health benefits to consumers and exhibit pumpable characteristics that can maintain high oxidative stability. High Oleic soybean oil (HOSO) is domestically produced in the US and typically contains low levels of saturated fat. In this study, HOSO was used to balance the saturated fatty acid profile of palm oil-based shortenings. 

The objective of this study was to investigate the use of high oleic palm oil types in the production of pumpable shortenings as an alternative to traditional palm oil. Texture and thermo-mechanical properties of the oils and shortenings were first assessed. Then, performance was compared through the measurement of the physical properties of high-fat cookies using the unblended oils and shortenings. 

## 2. Materials and Methods

### 2.1. Materials

The oils used in this study were traditional palm oil (TPO), obtained from Fuji Vegetable Oil, Inc. (Savannah, GA, USA); high oleic palm oil (HOPO) and high oleic red palm olein (HORO) produced by Del Llano Alto Oleico (Villavicencio, Meta, Colombia) and donated by Thin Oil Products, LLC. and High Oleic Soybean Oil (HOSO) donated by Pioneer^®^ (Ayer, MA, USA). Oils were produced no more than 6 months before the tests. They were stored at 4 °C in white, 20 L high density polyethylene (HDPE) jugs. Prior to use, each jug was heated to 40 °C for 1 h and gently mixed with an overhead propeller mixer. Aliquots of 1 L were transferred to glass mason jars, and each jar was nitrogen flushed before closing and string at 4 °C. These aliquots were used within 3 months of storage. Trancendim T130, a palm-based emulsifier comprised by saturated diglycerides (67%), triglycerides (27%) and monoglycerides (6%), was provided by Corbion Ingredients (Lenexa, KS, USA). The fatty acid composition of the oils and emulsifier used in this study are shown in Table 1.

To produce shortenings containing 30% (*w*/*w*) saturated fatty acids, HOPO, HORO, and TPO were individually combined with HOSO. The proportions of each oil in the shortenings are shown in Table 2. A concentration of 5% (*w*/*w*) emulsifier was used in all shortenings, which is the lowest usage suggested by the supplier to observe an effect in crystallization of the blends. 

### 2.2. Methods

The source of fat in the baked product was the independent variable of this study. This variable had three levels (1) HOPO-HOSO, (2) HORO-HOSO (3) TPO-HOSO. The null hypothesis (H0) was: There is no difference in physical properties of the cookies when using a HOPO-HOSO, HORO-HOSO or TPO-HOSO shortening. The dependent variables measured in each level were: (A) physical properties of shortenings: (i) Hardness, (ii) DSC profile (used in the calculation of SFC and melting points); and (B) physical properties of cookies: (iii) Hardness and fracturability, (iv) dimensions, (v) moisture, and (vi) water activity. No universal set of parameters have been established for a perfect cookie; however, these characteristics were used to compare the production batches so industry/researchers will be able to anticipate the effect of alternative oils on the physical properties of shortenings and baked goods.

#### 2.2.1. Shortening Preparation

A customized unit was developed for batch production of shortening in the Food Processing Laboratory at the University of Florida. It consisted of a thin-walled metal vessel jacketed with inlets and outlets for the heating and cooling fluids (water and propylene glycol respectively). A mixing paddle was designed in SolidWorks 3D CAD software, 3D-printed on chopped carbon fiber, and powered using Arduino microprocessor-controlled stepper motors. The main function of the paddle was to scrape the walls and bottom of the vessel to homogenize the fat blend during crystallization. 

The design of the process was based on industrial production of shortening as described by Ghotra et al. [4]. One-liter batches of shortening were prepared by heating the oil components under continuous mixing until they reached 70 °C with emulsifier addition at 60 °C. The blend was held at 70 °C for 10 min. The mixture was cooled down to 1 ± 1 °C in approximately 15 min with a constant scraping speed of 192 rpm. Shortening samples were tempered at 21 ± 1 °C for 5 days before analysis and use in the baking application. The fatty acid proportions of the shortenings are displayed in Table 3.

#### 2.2.2. Hardness of Fat

Firmness was evaluated on a TA.XT2 texture analyzer following the parameters described by Stable-Microsystems [30], using a 6 mm diameter cylindrical probe. Pre-test speed was 1 mm/s, test and post-test speeds were 2 mm/s, and distance of penetration was 12 mm. Shortenings were bottled in 20 mL screw-capped vials immediately after cooldown and tempered at 21 ± 1 °C for 5 days. Measurements were taken on day 6. Five samples of each oil and shortening were tested.

#### 2.2.3. Thermal Characterization of Fats and Shortenings Based on Differential Scanning Calorimetry (DSC)

i. DSC analysis

Differential scanning calorimetry was used to assess the thermo-mechanical properties of the oils, emulsifier and shortenings. The procedure followed the official AOCS method Cj 1-94 [1]. Oils were cooled from 80 °C at a rate of 10 °C/min to −60 °C (exothermic thermogram) and heated at a rate of 10 °C/min to 80 °C (endothermic thermogram). All samples underwent the same heating/cooling regime and were run in duplicate. TA Universal Analysis software was used to obtain peak and onset temperatures. Duplicates of the procedure per fat were performed.

Melting point of oils and shortenings was determined from DSC melting thermogram data using the method from [31]. In brief, onset and melting temperatures were analyzed with Thermal Advantage Universal Analysis Software, and the melting point was defined as the temperature in °C at the offset of the last melting peak.

ii. Calculated Solid Fat Content (SFC)

Solid fat content of oils and shortenings was calculated from the DSC data following the corrected method of Márquez et al. [32] from the original method of Kaisersberger, E. [33]. A straight baseline was drawn in the melting thermograms from the lowest onset melting temperature (T_0_) to the end of the last melting peak (T_f_) with axes of temperature in °C vs. heat flow in mW. Then, Equation (1) [32] was used to calculate melting enthalpy ΔH in mJ/mg as function of temperature T in °C.

(1) ΔH=98.13+1.62×T

Equation (2) [32] was used for the calculation of melted mass (m) in mg using the previously obtained ΔH. The melting energy (E) in mJ was defined as the area under the curve using the thermogram with axes of time in sec vs. heat flow in mW as shown in Equation (3). The percentage of melted mass at each temperature (T) was used in the calculation of the SFC as shown in Equation (4).

(2) $$m=\frac{E}{\Delta H}$$

(3) $$E=\int_{t_{0}}^{t_{f}}\mathrm{Heat}\;\mathrm{flow}\;\mathrm{dt}$$

(4) $${\mathrm{SFC}}_{m}=100-\frac{m_{T}}{\sum_{T_{0}}^{T_{f}}m}\times 100$$

#### 2.2.4. Cookie Preparation

Formulation of the cookies followed the AACC 10-53.05 official method [2] as shown in Table 4. This formula was deemed suitable for the study because fat content was similar to industrial formulations of wire-cut cookies in which firm fats are commonly used [34]. The fat portion of the formula was replaced by each of the studied oils HOPO, HORO, TPO, and their respective shortenings. Minor modifications were made to the AACC method to facilitate the handling of the dough as follows: After mixing, the dough was refrigerated until it reached 20 ± 1 °C. Twenty grams of dough were weighed and shaped with a cookie cutter and a plunger, resulting in disks of 5 cm diameter and 9 mm thickness. Twelve cookies (four per type of fat) were randomly placed on each tray. This ensured that all cookies underwent the same baking conditions. Four different batches were baked for each oil and shortening resulting in a total of eight trays (96 total cookies). Each tray was baked in a convection oven at 205 °C for 7 min individually. Cookies were removed from the oven and left to cool down for 40 min, then placed in sealed polyethylene bags and plastic containers for further analysis.

#### 2.2.5. Physical Properties of Cookies

i. Dimensions

Measures of diameter and thickness after baking followed the AACC official method [2]. According to the method, the average value of four orthogonal diameters of eight cookies lined side to side was recorded for each treatment. Thickness was assessed as the average height of eight cookies stacked on top of each other. Each treatment was performed in duplicate.

ii. Moisture and Water Activity

Moisture was determined according to the AACC 44-15.02 Air-oven method [35]. Cookies were selected 24 h after baking, then 2 g from each cookie were placed in moisture dishes and dried in an air-oven for 1 h at 130 °C. All moisture contents are reported on a percentage wet basis. Water activity (Aw) was measured using a water activity meter (Aqualab 4TE DUO). Triplicate measurements were taken per treatment.

iii. Hardness and Fracturability

Texture analyses were performed using an XT Plus texture analyzer (Texture Technologies Corp., Scarsdale, NY, USA) equipped with a three-point HDP/3PB bend rig with a rounded edge blade. The cookies were held in the center of the supports 30 mm apart. The speed of the test was 3 mm/s, with pre- and post-test speeds of 1 mm/s and 10 mm/s, and a load cell of 50 kg. Maximum force (gf) applied during braking was the measure of cookie hardness. Fracturability was recorded as the distance (mm) traveled by the rig blade at the first breakpoint. Eight cookies per type of fat were analyzed.

#### 2.2.6. Statistical Analysis

Data are presented as means and standard deviations. Comparisons between cookies were tested with 1-way ANOVA with type of fat as factor, and mean separation was done with Tukey’s HSD test. Significance level for all tests was α = 0.05. Data analysis was performed using Prism 8 (GraphPad Software, Inc., San Diego, CA, USA).

## 3. Results and Discussion

### 3.1. Hardness of Oils and Shortenings

**Figure 1:**
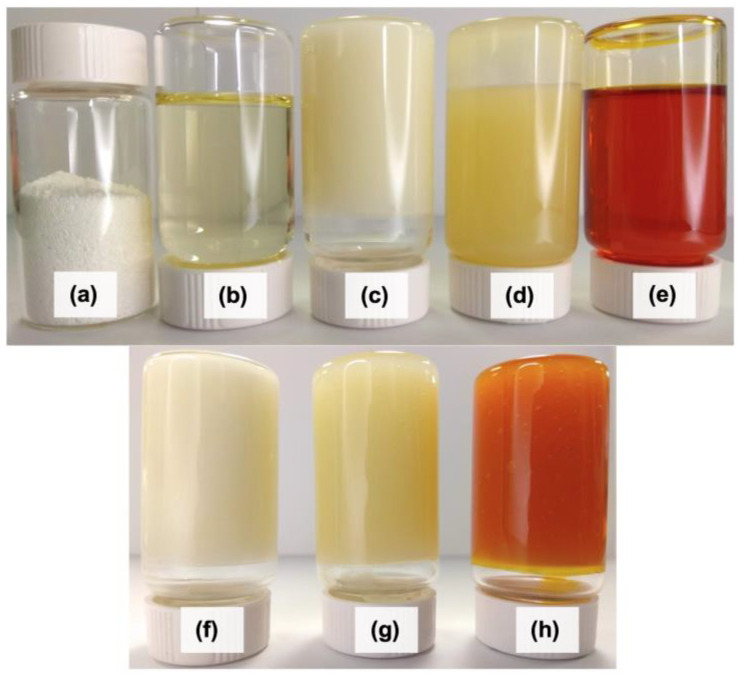
Appearance of oils, emulsifier and produced shortenings. (**a**) T130 emulsifier, (**b**) HOSO, (**c**) TPO, (**d**) HOPO, (**e**) HORO, (**f**) TPO shortening, (**g**) HOPO shortening, (**h**) HORO shortening.

The appearance of the oils relative to the shortenings is displayed in Figure 1. Inverted containers show the capacity of the shortenings to remain suspended in the bottom of the vials for 35 min at 21 ± 1 °C, contrary to the oils, which flowed easily. 

TPO was the only oil with measurable hardness (128.0 gf) and work of penetration (576.7 gf·s) at room temperature, which was expected due to its higher level of saturated fat solid at room temperature compared to HOPO and HORO. HOPO and HORO showed <5 gf hardness at room temperature, while their shortenings exhibited low but detectable hardness (10 gf), as shown in Table 5.

The aforementioned results suggest that equal amounts of saturated fat and structuring agent were not the only factors needed to produce shortenings with similar hardness. Properties such as triglyceride composition of the fat and the resulting crystalline fat structure of the shortenings may be additional factors that play a major role in hardness. These links between hardness and thermal properties of the fats, complemented with previous literature are provided in the next sections. However, additional study on the crystallization profile of HOPO, HORO, and their shortenings would be needed to adjust the processing parameters that allow tailoring the final hardness of the shortenings.

### 3.2. Thermal Characterization of Fats and Shortenings Differential Scanning Calorimetry (DSC) 

#### 3.2.1. DSC Analysis

**Figure 2:**
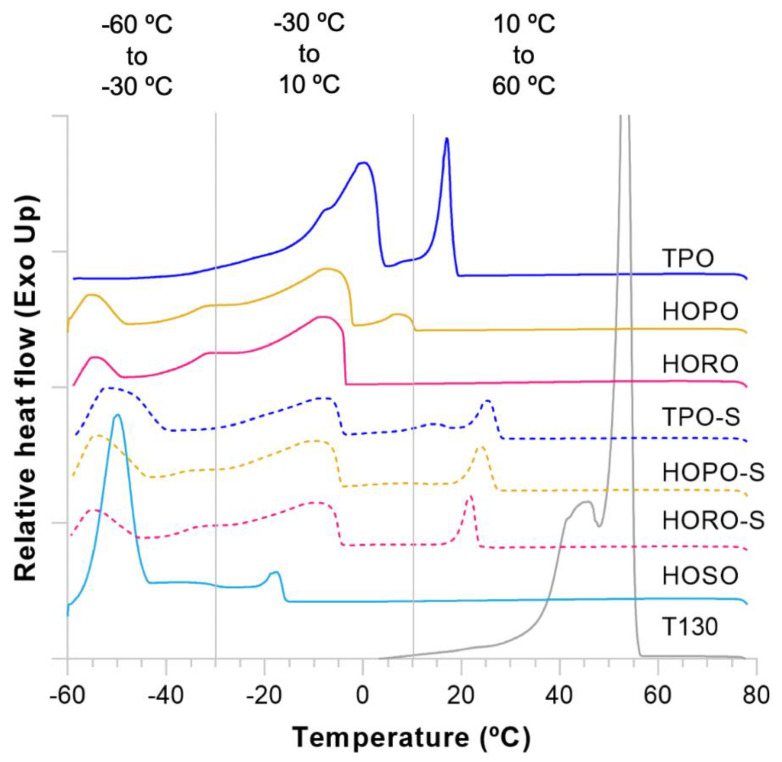
Differential scanning calorimetry of crystallization curves (exothermic thermogram) for oils, emulsifier and shortenings. Deviations above the baseline are exothermic; Deviations below the baseline are endothermic.

**Figure 3:**
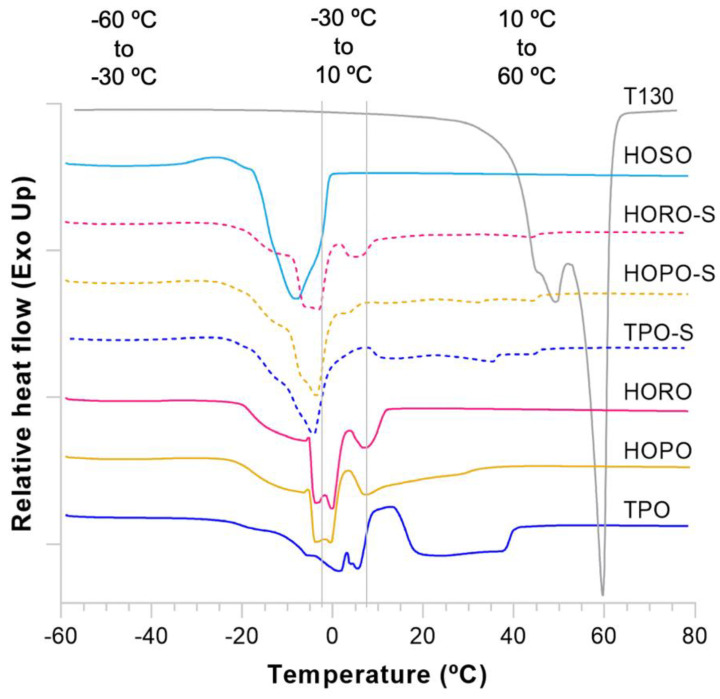
Differential scanning calorimetry of melting curves (endothermic thermogram) for oils, emulsifier and shortenings. Deviations above the baseline are exothermic; Deviations below the baseline are endothermic.

DSC thermograms were collected for each oil, emulsifier and shortening to identify temperature regions at which phase and polymorphic transitions take place (Figure 2 and Figure 3). The onset and peak temperatures, and the enthalpy of melting and crystallization are shown in Table 6 (crystallization) and Table 7 (melting). TPO showed two exothermic peaks (Figure 2) which have been reported by several researchers [36,37], a sharp peak at 21.3 °C related to a high melting point fat fraction (stearin) and a dispersed group of peaks with the highest peak temperature at 1.9 °C pertaining to a low melting point fraction (olein) [38]. Two peaks were also observed in HOPO, a smaller first peak at 6.4 °C and a second set with a highest temperature of −6.2 °C. The smaller size of the first exotherm in HOPO is likely related to a lower proportion of stearin vs. olein of 20:80 in HOPO compared to 35:65 in TPO [39].

The structure of fats is characterized by the organization of their components, which in most vegetable fats are triglycerides. The most abundant triglycerides by mass percentage in TPO are PPO (27.4%), POO (21.4%) and PLO (10.0%) [40], while the most abundant in HOPO are POO (32.6%), PPO (20.4%), and OOO (10.7%) [25], with P (palmitic acid), O (oleic acid) and L (linoleic acid) the fatty acids involved. To understand effects of triglyceride composition and the observed DSC profiles of shortenings, the melting temperatures of these triglycerides (in their most stable polymorph) are key, being 35 °C for PPO [41], 12–15 °C for POO, and 10.7 °C for OOO [40]. As mentioned by Narine and Marangoni [42], the first sharp exothermic peak in TPO indicates the presence of triglycerides that are good templates for crystallization, which leads to formation of uniform and organized crystals during cooling. The di-saturated triglycerides that dictate the first nucleation pattern then contribute to uniformity of the crystal networks, which has been strongly positively correlated to harder fat structures [43,44]. Conversely, HOPO contains a major fraction of tri-unsaturated triglycerides, which have a highly amorphous arrangement upon crystallization and presumably form less strong crystal networks. These microstructural differences are likely the main contributors to the differences in size, shape, location of exotherms and a higher hardness in TPO oil and shortening compared to HOPO and HORO oils and shortenings. 

All shortening crystallization thermograms showed an accentuated separation of two exothermic peaks (Figure 2). For the shortenings of TPO, HOPO, and HORO, the peak temperatures of the first exotherms were recorded at 25.2 °C, 24.2 °C, and 20.4 °C respectively. All peak temperatures of first exotherms were higher than their respective peak temperatures of unblended oils. The increase in the temperature of the first exotherm is linked to monoglyceride and diglyceride addition, which registered a peak temperature in the first exotherm of 53.4 °C. These compounds modify the nucleation stages of crystallization as well as the size of the crystals in fats [42], acting as homogenizers of onset crystallization temperatures. The fact that all shortenings had a similar offset of the last endotherm (Figure 3) at 45.9 °C, 45.9 °C, and 45.3 °C for TPO, HOPO, and HORO shortenings respectively is also a consequence of the emulsification. However, the newly formed exotherms in the shortenings with the addition of emulsifiers are small and broad, which suggests that the emulsifier used does not act as a strong crystal formation promoter, and different types of nucleation templates were formed in all shortenings [45].

An intermediate exotherm with peak at 14.3 °C was only observed in TPO shortening, which indicates the presence of mid-melting triglycerides in TPO that are not present in HOPO or HORO. This is also reflected in a deeper endotherm of TPO shortening (Figure 3), with onset temperature of 9.3 °C compared to flatter endotherms of HOPO and HORO with onset temperatures of 8.6 °C and 4.1 °C, respectively. The differences in heat flow of the last endotherm suggest that more mid-melting triglycerides were available in TPO shortening to keep their stable crystal forms after the onset melting temperature compared to HOPO and HORO shortenings.

The melting points of unblended oils determined by DSC agreed with literature values obtained by different methods for TPO, HOPO and emulsifier as shown in Table 8. Thus, it was confirmed there was an elevation in the melting point compared to the base oils for all shortenings; Higher melting points, together with higher SFC at room temperature, contributed to higher hardness of the fats at room temperature. This correlation between melting point and hardness was previously observed by [46,47] when they studied the addition of saturated triglycerides in fat blends. Increase in melting points were also observed by Ng et al. [48] who recorded new thermal transitions at higher temperatures in blends of superolein with diglycerides as a structuring agent. Authors such as [49] compiled studies in which the addition of saturated monoglycerides and diglycerides increased the melting points, accelerated the crystal growth of vegetable oils, and acted as modifiers of the crystal structure of fats. The newly formed exotherms in the shortenings with the addition of emulsifiers are small and broad, which suggests that the emulsifier used does not act as a strong crystal formation promoter, and different types of nucleation templates are formed in all shortenings [45]. However previous studies on the effect of saturated diglycerides in palm oils have shown opposite effects depending on the location of the fatty acids in the diglyceride. Decrease in melting point and delay in crystal formation was observed with the 1,2 isomer while the 1,3 isomer at levels of 5% increased melting point of palm oil and stearin [50].

Although melting points below body temperature are indicated to reduce waxy mouthfeel of the products containing them [46], SFC also dictates the expected performance of the fat in the product. Thus, even when the melting point of all shortenings was around 45 °C, the percent solids suspended at body temperature was below 3% in TPO, HOPO and HORO shortenings, which prevented unpleasant mouthfeel [51]. Karabulut and Turan [52] reported melting points of 25 commercial margarines and shortenings ranging from 21.9 °C and 49.2 °C, placing the shortenings of TPO, HOPO and HORO within commercial limits. A consequence of high melting points combined with low SFC is that the fat blends pump more easily. Fats that have characteristics of liquid shortenings, with SFC 10–15% at 20 °C can be easily pumped and bottled, unlike conventional plastic shortenings [46].

#### 3.2.2. Solid Fat Content (SFC) 

**Figure 4:**
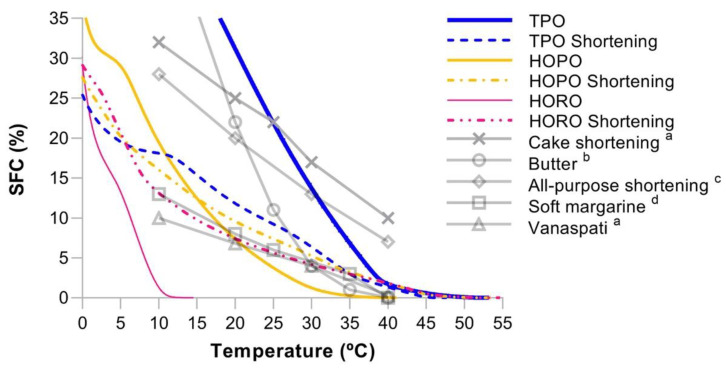
Solid Fat Content as calculated using DSC measurements for TPO, HOPO and HORO shortenings/oils. Literature SFC for commonly used baking fats are provided for comparison purposes and are represented as greyed values. Sources: ^a^ [55], ^b^ [56], ^c^ [13], ^d^ [4]. Abbreviation—S: Shortening.

As shown in Figure 4, SFC curves of individual oils exhibited steeper slopes compared to SFC of shortenings. Flatter slopes have been correlated to higher diversity of fatty acids within triglycerides, which causes a better distribution of melting events over a wider temperature range [33]. The curves of shortenings from HOPO and HORO had an offset below the TPO shortening curve, which correlates with the smaller peaks in their DSC endotherms (Figure 3). Strong correlation between SFC and hardness of fat has been previously observed [18,53] to the extent that SFC can be used as a proxy variable to estimate characteristics like spreadability, heat tolerance power and usage temperature. Narine & Marangoni [54] explained how the shear elastic modulus, the resistance of fat to tangential shear before deformation, is positively correlated to SFC. They showed that crystalline structures are the only building blocks larger than triglycerides, and they significantly contribute to the mechanical strength of fat crystal networks [42]. 

The SFC curves of the produced shortenings exhibited a behavior that has been linked to desirable attributes of fats. Teles Dos Santos et al. [57] listed some positive attributes with which HOPO and HORO shortenings are consistent, such as SFC <32% in the range of 4–10 °C to facilitate shortening workability at refrigerator temperatures and SFC < 1–3% in the range of 35–37 °C to avoid waxy mouthfeel. A parameter of SFC >10% between 20–22 °C to maintain the integrity of the product and avoid oil exudation was only exhibited by TPO and HOPO shortenings, which registered 10–12% and 8–10% SFC respectively at that temperature range compared to 6–7% for HORO shortening. This indicates that the TPO, HOPO and HORO shortenings described in this research can be useful as fluid or pumpable shortenings at room temperature. SFC curves from TPO, HOPO and HORO shortenings were similar to commercially available fats as displayed in Figure 3. These fats include soft margarine [4]; hydrogenated fat blends such as vanaspati [55] -a commonly used fat for frying and baking as a replacement of butterfat- [3]; and similar to blends that combine fully hydrogenated fat with interesterified oils, as compiled by Wassell and Young [58]. 

### 3.3. Physical Properties of Cookies

i. Dimensions of cookies

**Figure 5:**
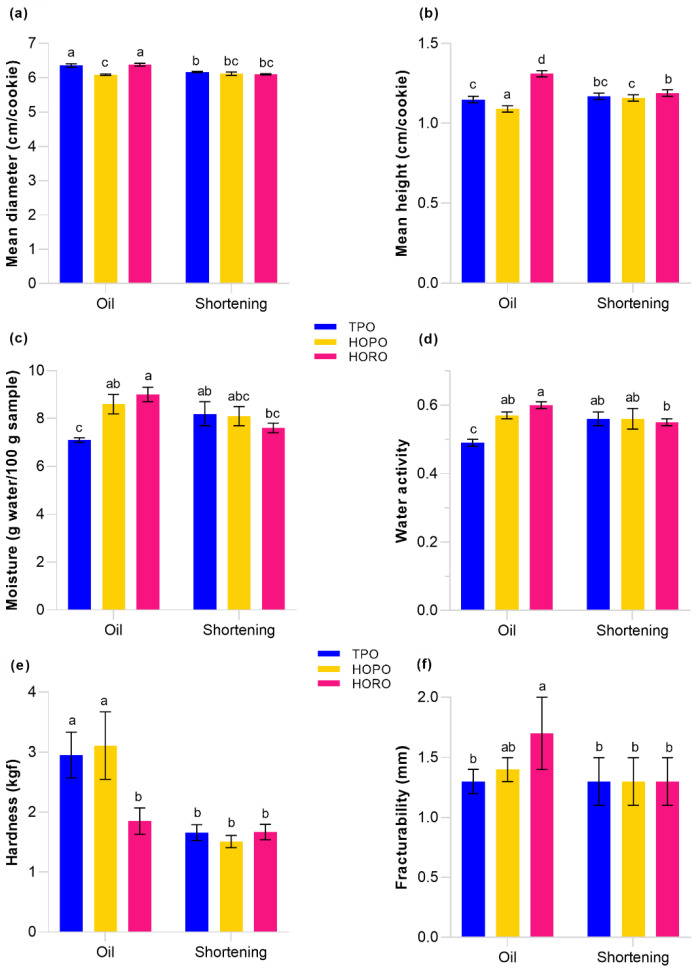
Physical properties of cookies made with oil and shortenings. Different letters above the bars indicate statistically significant difference at *p* ≤ 0.05 (one-way ANOVA). (**a**,**b**) Mean diameter and height: A standard deviation of 0.02 cm represents the smallest measurement that could be reliably taken per cookie. *n* = 2 for all measurements; (**c**,**d**) Moisture and water activity: *n* = 3 for all measurements; (**e**,**f**) Hardness and fracturability: *n* = 8 for all measurements.

The diameter and height of cookies made with both oils and shortenings are shown in Figure 5a,b, respectively. Cookies with HORO oil had the largest increase in height from the initial dough height of 0.9 cm. There was a significant difference between diameter and height of cookies made using unblended oils, but variation was not proportional to saturated fatty acid content. The low correlation between fat saturation and cookie dimension is also reflected in the literature; some studies have found saturated fatty acids increased the volume of cookies by creating interactions with the components of dough during the mixing process, which leads to better trapping of air [59]. Other studies have correlated saturated fat content with little or no change in cookie height values [60,61], while it has been shown that liquid oils are able to delay the set point of the dough, which allows a higher spread or diameter during baking before the matrix hardens [62]. In this study, there were similarities in dimensions of cookies made with TPO, HOPO and HORO shortenings, which contained the same saturated fat percentage, resulting in mean diameter and height of 6.1 cm and 1.2 cm respectively, suggesting that similar fatty acid compositions can result in similar effects on cookie volume, however, other properties of the fats such as triglyceride composition or melting point may also contribute to the similarity of dimensions.

ii. Moisture Content and Water Activity

Cookie moisture content and water activity results are shown in Figure 5c,d. Cookie dough had a moisture content 17 g water per 100 g of dough and water activity of 0.82 with no significant difference between mean values of moisture content and water activity of the six dough samples before baking. 

Cookies produced with HOPO and HORO oil retained on average 1.5 g and 2 g more water than TPO cookies respectively (compared on a 100 g basis). A similar trend was observed in TPO shortening cookies which retained on average 1.1 g more water compared to TPO oil cookies. Based upon these findings, an increase in saturated fat content appeared to be negatively correlated with moisture content. Water activity followed a similar behavior to moisture content, with lower values recorded at higher levels of saturated fat. 

There were no significant differences in moisture content or water activity between cookies made with shortenings. These results are in agreement with [26] in which it was observed that higher contents of saturated fat in palm olein were highly correlated with lower water activity and moisture values even at different baking regimes of cookies. This has been explained by [59] as an effect of the presence of crystalline lipids that effectively coat the starch granules and lower water absorbed by the dough.

iii. Hardness and Fracturability of Cookies

Results for hardness and fracturability of all cookies is shown in Figure 5e,f. Cookies made using TPO and HOPO showed no significant differences in hardness and fracturability mean values of 3 kgf and 1.37 mm respectively. This similarity in hardness and fracturability despite the difference in saturated fatty suggests there is a commonality in the interaction of TPO and HOPO with the components of the dough that are responsible for cookie structure, mainly gluten and sugar, which does not depend solely on their fatty acid composition.

There was a convergence of hardness to 1.6 kgf in all shortening cookies, which correlated with a convergence in values in moisture and water activity. Constant emulsifier concentrations combined with equally saturated fat content in shortening cookies may have exerted an effect of homogenization in moisture migration during baking. Authors such as Pareyt et al. [59], Goldstein et al. [60] have also confirmed a softening effect of emulsified shortenings on cookie crumb. Jadhav et al. [17] obtained softer cookies made with an ethyl cellulose-emulsified medium chain triglyceride (MCT) oleogel compared to the MTC alone, explained as a result of the emulsifier acting as air-trapping aid during creaming. The softer cookies resulted in higher consumer preference. The fat percentage in the formulation and the type of cookie may also determine the role played by saturated fatty acids, more evidently during shelf-life. The study by Giuffrè et al. [63] found an inverse correlation between water activity and both hardness and fracturability over 12-month storage. Compared to our results in fresh cookies, there was no clear correlation between water activity and hardness or fracturability, evident trends may highlight after the first month of production.

This study compared the physical properties of shortenings and cookies made with different sources of fat while controlling for the saturated fatty acid content. Significant differences were observed that can be applied by industry in product design. To better understand the mechanisms involved, future work will need to include rheological studies, characterization of the types of crystals formed, triglyceride composition, and sensory studies utilizing high oleic oils and shortenings.

## 4. Conclusions

Three emulsified shortenings were produced using different palm oil types, HOPO, HORO and TPO, under identical processing conditions. They were formulated to contain 30% *w*/*w* saturated fat, using HOSO to balance the composition, and with equal emulsifier and structuring agent concentrations. HOPO and HORO shortenings exhibited lower hardness at room temperature compared to TPO shortening. This was correlated with inherent characteristics of the unblended oils, such as the absence of mid-melting peaks observed via DSC, and lower SFC of the high oleic oils compared to TPO. Despite thermal and physical differences recorded between the three shortenings, there were no significant differences between dimensions, moisture content, water activity, hardness and fracturability of cookies made with them. 

It was demonstrated in this study that two high oleic palm oil fractions (HOPO and HORO) are viable alternatives to currently used saturated fats such as TPO, resulting in similar physical properties in final products. High oleic shortenings could be explored as replacement of hydrogenated fats, and/or interesterified fats in pumpable shortening production, with an array of potential applications within the baking industry. These shortenings could also serve in traditional recipes as a potential fortifying ingredient of bioactive components such as pro-vitamin A carotenes and vitamin E tocols, without altering the physical characteristics of baked products.

## Figures and Tables

**Table 1:** Fatty acid composition (% *w*/*w*) of fats and oils used in this study.

	Oils (% *w*/*w*)				
Fatty Acids (FA)	TPO	HOPO	HORO	HOSO	T130
C8:0		0.01			
C10:0		0.02			
C12:0	0.2	0.2	0.3		0.2
C14:0	1.0	0.4	0.4	0.0	1.0
C15:0	0.1	0.1	0.1	0.0	0.1
C16:0	42.3	27.8	25.9	6.1	52.9
C17:0	0.1	0.1	0.1	0.7	0.2
C18:0	4.2	2.8	2.7	4.1	44.0
C20:0	0.4	0.3	0.3	0.4	0.5
C22:0	0.1	0.1	0.1	0.4	0.1
C24:0	0.1		0.1		0.1
C16:1	0.2	0.3	0.3	0.1	
C18:1	41.0	55.5	56.8	76.0	0.6
C20:1	0.2	0.1	0.2	0.3	
C18:2	10.0	11.9	12.6	9.3	0.3
C18:3 (α)	0.2		0.3	2.4	
C18:3 (γ)		0.2			
Saturated FA	48.5	31.8	29.8	11.8	99.0
Monounsaturated FA	41.3	56.0	57.3	76.4	0.6
Polyunsaturated FA	10.1	12.2	12.8	11.7	0.3
Other FA	0.1	0.1	0.1	0.1	0.1

FA composition analysis was performed by FoodChain ID Testing Laboratories. HOPO: High oleic palm oil. HORO: High oleic red palm olein. TPO: Traditional palm oil. HORO: High oleic soybean oil. T130: Mono- and di- glyceride emulsifier. Other FA include saturated, mono- and poly- unsaturated fatty acids which were not detected by the AOCS Ce -1b-89 method as reported by FoodChain ID Testing Laboratories.

**Table 2:** Percent content of fats used in formulation of shortenings as (% *w*/*w*).

	Oils (% *w*/*w*) *				
Shortenings	TPO	HOPO	HORO	HOSO	T130
TPO shortening	36.7			58.3	5.0
HOPO shortening		66.1		28.9	5.0
HORO shortening			79.6	15.4	5.0

* % *w*/*w*: g of oil/100 g of shortening.

**Table 3:** Calculated fatty acid proportions (% *w*/*w*) of produced shortenings.

	Fatty Acids (% *w*/*w*) *		
Fat	Saturated FA	Monounsaturated FA	Polyunsaturated FA
TPO shortening	29.7	59.7	10.5
HOPO shortening	29.4	59.1	11.4
HORO shortening	30.5	57.4	12.0

* Values were calculated from the original fatty acid composition of the oils and emulsifier.

**Table 4:** Composition of cookie dough samples.

Ingredient	% *w*/*w* in the Formulation
Wheat flour	47.8
Sucrose	20.1
Fat *	19.1
Corn syrup (30 °Bx)	0.7
Skimmed powdered milk	0.5
Ammonium bicarbonate	0.2
Sodium bicarbonate	0.5
Water	10.5
Salt	0.6

* Fat was replaced by each of the oils (TPO, HOPO, HORO) and their respective shortenings tested in the study.

**Table 5:** Hardness (gf) and work of penetration (gf·s) of produced shortenings.

Fat	Hardness (gf)	Work of Penetration (gf·s)
TPO	128.0 ± 2.4	576.7 ± 22.2
TPO shortening	33.0 ± 3.5	106.7 ± 21.5
HOPO	<5	<5
HOPO shortening	10.1 ± 1.8	<5
HORO	<5	<5
HORO shortening	10.0 ± 1.7	<5

Note: 5 gf was the limit of detection for this test. Data is presented as mean ± standard deviation. *n* = 5 for all measurements.

**Table 6:** Crystallization onset and peak temperature (°C), and enthalpy of fats ∆H (J/g).

Fat	Temperature Ranges						∆H (J/g)
	60 °C to 10 °C		10 °C to −30 °C		−30 °C to −60 °C		
	T Onset	T Peak	T Onset	T Peak	T Onset	T Peak	
TPO	18.6 ± 0.2	17.3 ± 0.3	3.8 ± 0.1	0.5 ± 0.4			42.5 ± 0.7
HOPO			9.9 ± 0.8	7.7 ± 0.3	−48.7 ± 0.1	−54.8 ± 0.3	31.8 ± 0.9
			−2.1 ± 0.01	−7.3 ± 0.1			
HORO			−3.5 ± 0.01	−7.5 ± 0.1	−49.1 ± 0.4	−54.8 ± 0.1	32.5 ± 0.4
HOSO			−15.8 ± 0.01	−17.4 ± 0.1	−44.8 ± 0.1	−49.9 ± 0.01	31.6 ± 0.7
T130	55.6 ± 0.1	53.4 ± 0.2					96.8 ± 0.01
	48.6 ± 1.1	46.6 ± 0.1					
TPO-S	27.7 ± 0.3	25.2 ± 0.3	−4 ± 0.01	−7.4 ± 0.4	−40.6 ± 0.01	−52.3 ± 0.1	34.3 ± 1.3
	17.5 ± 0.2	14.3 ± 0.2					
HOPO-S	27.1 ± 0.2	24.2 ± 0.1	−4.4 ± 0.01	−8.6 ± 0.4	−44.2 ± 0.1	−54.5 ± 0.01	29 ± 0.9
HORO-S	22.3 ± 1.6	20.4 ± 2	−4.3 ± 0.2	−8.9 ± 0.3	−46.7 ± 0.2	−54.8 ± 0.2	29.1 ± 0.9

Note: Abbreviation—S for shortening. Fats with multiple lines represent all peaks in the corresponding temperature range. Reported in order of appearance from right to left in the crystallization curve. *n* = 2 for all measurements. A standard deviation of 0.01 °C represents the smallest measure that could be reliably taken by the equipment.

**Table 7:** Melting onset and peak temperature (°C), and enthalpy of fats ∆H (J/g).

Fat	Temperature Ranges						∆H (J/g)
	−60 °C to 0 °C		0 °C to 10 °C		10 °C to 60 °C		
	T Onset	T Peak	T Onset	T Peak	T Onset	T Peak	
TPO	−25.2 ± 1.6	1.9 ± 0.2	15.5 ± 1.5	21.3 ± 1.5			77.3 ± 2.6
HOPO	−21.3 ± 0.01	−6.2 ± 0.01	3.5 ± 0.02	6.4 ± 0.1			54.6 ± 0.1
	−5.5 ± 0.02	−3.8 ± 0.01					
	−1.5 ± 0.01	−0.4 ± 0.01					
HORO	−30 ± 0.3	−5.8 ± 0.3	4.1 ± 0.2	7.3 ± 0.4			48.6 ± 0.01
	−5.4 ± 0.1	−3.6 ± 0.01					
	−1.5 ± 0.01	−0.2 ± 0.01					
HOSO	−26.5 ± 0.7	−7.9 ± 0.05					57.1 ± 0.3
T130					30.1 ± 0.1	49.6 ± 0.2	96.7 ± 0.01
					53.1 ± 1	59.7 ± 0	
TPO-S	−24.2 ± 2.2	−3.9 ± 0.5			9.3 ± 1.5	35.1 ± 0.8	64.7 ± 2.3
HOPO-S	−25.8 ± 1.5	−3.5 ± 0.2			8.6 ± 2.7	32.2 ± 0.8	59 ± 2.4
HORO-S	−28.2 ± 0.2	−2.8 ± 0.3	2.3 ± 0.1	6 ± 1.2	32.4 ± 0.3	44.4 ± 0.2	55.4 ± 2.3

Note: Abbreviation -S for shortening. Fats with multiple lines represent all peaks in the corresponding temperature range. Reported in order of appearance from left to right in the melting curve. *n* = 2 for all measurements. A standard deviation of 0.01 °C represents the smallest measure that could be reliably taken by the equipment.

**Table 8:** Melting points (°C) of unblended oils, emulsifier and produced shortenings.

Fat	Melting Point (°C)	Literature Values (°C)	Reference
TPO	39.9 ± 0.2	35–37	[31]
HOPO	30.5 ± 0.9	26–28	[22]
HORO	11.1 ± 0.5	12–16	[22]
HOSO	−0.8 ± 0.0	N/A	-
T130	61.8 ± 0.3	60–63	Corbion Ingredients
TPO Shortening	45.9 ± 0.1		
HOPO Shortening	45.9 ± 0.1		
HORO Shortening	45.3 ± 0.8		

N/A: Not available. Data is presented as mean ± standard deviation. *n* = 2 for all calculated values.

## Data Availability

Data is contained within the article.
